# Can Executive Functions and Severity of Attention Deficit Hyperactivity Disorder Predict Symptom Levels of Developmental Coordination Disorder in Children with Attention Deficit Hyperactivity Disorder? a Preliminary Study

**DOI:** 10.1192/j.eurpsy.2025.845

**Published:** 2025-08-26

**Authors:** F. U. Dundar, H. A. Guler

**Affiliations:** 1 Child and Adolescent Psychiatry, Selcuk University, Konya, Türkiye

## Abstract

**Introduction:**

Attention Deficit Hyperactivity Disorder (ADHD) is a childhood-onset disorder characterized by symptoms of attention deficit, hyperactivity and impulsivity, and executive functioning difficulties. Developmental Coordination Disorder (DCD) is a neurodevelopmental disorder that involves motor coordination problems with frequent difficulties in movements that most typically developing children can easily accomplish. The most common comorbidity accompanying DCD is ADHD. It has been reported that DCD frequently accompanies ADHD even if it is not at the diagnostic level and may cause loss of functionality in daily life. In this study, we aimed to investigate the relationship between DCD symptoms and ADHD severity and executive functions in children and adolescents with ADHD without a diagnosis of DCD.

**Objectives:**

The study sample consisted of children aged 7-15 years with ADHD who were evaluated in the outpatient clinic of the Department of Child and Adolescent Psychiatry at Selçuk University. Children with medical conditions requiring physical therapy, neurological disorders, DCD, tic disorders, movement disorders, autism spectrum disorder, and intellectual disability were excluded from the study.

**Methods:**

The Turgay Disruptive Behavior Disorders Screening and Evaluation Scale based on DSM-IV was used to determine the severity of ADHD and the Stroop Test was used to evaluate executive functions. Revised Developmental Coordination Disorder Battery was applied for the symptoms of DCD. Ethics committee approval for the study was obtained from Selçuk University Faculty of Medicine Local Ethics Committee. (2024/411)

**Results:**

15 girls and 19 boys between the ages of 7-15 participated in the study. The mean age was 10.71±2.83 years. A significant correlation was found between patients’ Turgay attention subscale (p:0.010), oppositional defiant subscale (p:0.027) and conduct disorders subscale (p:0.028) scores and DCD symptom levels. No correlation was found between Stroop test results and DCD symptom levels.

**Conclusions:**

In this study, the relationship between ADHD severity, executive functions and symptoms of DCD in children and adolescents diagnosed with ADHD was examined. The findings indicate that ADHD severity and DCD symptoms may be related in individuals with ADHD without a diagnosis of DCD. This may indicate that in individuals diagnosed with ADHD, DCD symptoms may be related to ADHD severity rather than executive functions. The fact that the study was single centered and the sample size was small limits the generalizability of the findings. Studies with larger samples are needed.

**Disclosure of Interest:**

None Declared

